# The Effects of High-Altitude Environment on Brain Function in a Seizure Model of Young-Aged Rats

**DOI:** 10.3389/fped.2020.00561

**Published:** 2020-09-10

**Authors:** Yao Xie, Shenglan Qin, Rui Zhang, Hong Wu, Guoyu Sun, Lili Liu, Xinlin Hou

**Affiliations:** ^1^Pediatric Department, Peking University First Hospital, Beijing, China; ^2^Pediatric Department, People's Hospital of Tibet Autonomous Region, Tibet, China

**Keywords:** high altitude, seizures, brain function, hypoxia, young-aged rats

## Abstract

In this study, we examined the effects of high-altitude environment on the brain function of a young-rat seizure model. Two-hundred healthy, 3-week old, male rats were selected and equally divided into the plateau and plain groups. The plateau group was preconditioned in a simulated 5,000-m altitude (barometric pressure [PB], 405 mmHg; partial pressure of oxygen [PO_2_], 84 mmHg) for 6 h/day for 7 days, while the plain group was kept in the ordinary atmospheric environment (PB, 760 mmHg; PO_2_, 157 mmHg) for 7 days. After preconditioning, rats were administered pentylenetetrazol (PTZ) to generate level-4 or stronger seizures. Electroencephalogram (EEG) signals were recorded (16 rats/group); the histology and apoptosis of hippocampal tissue were evaluated (6 rats/group); and spatial learning and memory were examined in the Morris water maze (12 rats/group; 6-weeks old). To induce a level 4 or stronger seizure successfully, a significantly higher PTZ dose was used in the plateau (81.32 ± 21.57 mg/kg) than in the plain group (63.41 ± 19.77 mg/kg, *p* < 0.01); however, the plateau group survival rate was significantly lower than that of the plain group (26.2 vs. 42.9%, *p* < 0.05). EEG parameters did not differ between the two groups. Histological analysis revealed that in the plateau group, more neurons were observed (*p* < 0.001), especially in DG and CA1 areas, and less apoptotic cells were found in DG areas (*p* = 0.035), comparing with the plain group. No differences were found between the two groups in any of the parameters examined in the Morris water maze. Our results show that the disease outcome caused by low pressure and low oxygen environment in the plateau group was different to that in the plain group. The high drug dosage to induce seizures in the plateau group, accompanied by increased mortality rates after seizures, indicates that the seizure threshold may be higher in the plateau than in the plain group. Moreover, based on the histological findings, the plateau environment seems to exert a protective effect on brain development after seizures only for survived individuals with mild conditions.

## Introduction

Tibet is an area with an average altitude of about 4,000 m, with a pressure and partial pressure of oxygen significantly lower than those in plain areas. Tibet's special geographical state not only results in differences between disease spectra but also between the physiological states within the same disease. During one of our author's work as aid for Tibet, she found that for the young children especially the neonates with convulsion in Tibet, the outcome of survival children seemed to be better than in plain areas. It has been established that sustained severe hypoxia can lead to brain hypoxia, ischemia, and edema, eventually leading to neuronal necrosis and irreversible brain damage (1). However, recent studies suggest that moderate hypoxia may have a protective effect on brain injury-related diseases. To train pilots, the concept of “hypoxic training” was proposed as early as the 1930s (2). It is believed that moderate hypoxia training can increase the tolerance of human body, especially that of the cardiovascular system, to subsequent hypoxia. The training method can be a stay at high altitude camps for several weeks, regular high-altitude flights by airplane, training in altitude chambers, and inhalation of a low-oxygen mixture. Follow-up studies found that the low-pressure hypoxic preconditioning can improve other organs' tolerance to hypoxic-ischemic injury; this effect is also encountered in the brain, providing protection to cerebral ischemia and neurodegenerative diseases (3, 4). However, the degree to which hypoxia can induce damage or protection, and against which degree of brain injury hypoxia can provide protection is not clear and is still controversial. Therefore, the impact of plateau environment on diseases needs to be further studied.

Convulsions are common neurological symptoms in childhood and can be caused by a variety of reasons (5). The infantile, especially the neonatal period, is a critical period for nervous system development; thus, the convulsion-induced damage to the nervous system during this period can be considerable. The severity and duration of convulsive seizures are crucial factors influencing the outcomes. A study showed that in 89 patients, 23 (36%) had neurological abnormalities upon examination without an apparent functional impact, neonatal mortality due to convulsive seizures in term infants has been reported at 7%; while 28% of the survivors have poor long-term outcomes. Among the infants with overall poor neurological outcomes (25 patients), 20 (80%) had severe and 5(20%) had moderate neurological impairment (6). Recurrent epileptic seizures cause neuronal loss in the developing brain through apoptosis and necrosis and induce progressive memory deficits (7). In order to reduce those poor outcomes, many studies have been conducted to search for brain protective measures. Research on preconditioning has resulted in various promising strategies for the treatment of patients with acute brain injury (8). Among them, low-pressure hypoxic preconditioning has been widely studied. A study by Zhen et al. (9) suggested that moderate hypoxic preconditioning after epilepsy had a protective effect on brain injury. However, there is paucity of studies examining whether these protective effects are present at different hypoxic levels.

The Tibetan plateau provides a natural low-pressure hypoxic environment. The characteristics of children's convulsion in Tibet are special in outcomes compared with those in plain areas. However, it is still not clear whether the low-pressure hypoxia provided by the plateau environment leads to protection or damage as relevant research is lacking. And if the convulsions are severe, whether the low-pressure hypoxia plays a role is also a problem we want to clarify. To better understand the effect of the plateau environment on the severe convulsion and prognosis, we generated a convulsion rats model with sever levels (level 4–5). First, we preconditioned rats in a low-pressure hypoxia oxygen environment to simulate the plateau environment. Next, we monitored the rats' brain electrical activity, observed the pathological changes of brain tissues, and analyzed the results of the Morris water maze experiment to evaluate the effects of low-pressure hypoxia on brain function.

## Materials and Methods

### Animals

All animal experiments were approved by the local Animal Welfare Committee. We randomly divided 200 3-week-old (the earliest time to withdraw breastfeeding is 21 days after birth), healthy male, Sprague–Dawley rats (weight 60–80 g) into an experimental group (100 rats), and a control group (100 rats). We chose male rats to exclude any effects on epilepsy produced by estrogens or the estrous cycle of female rats (10). The animal experimental protocols were performed in accordance with the Animal Management Rule of the Chinese Ministry of Health and the Animal Care Committee of Peking University First Hospital, Beijing, China. All rats were born and raised at Beijing Vital River Laboratory Animal Technologies Co., Ltd., for the first 21 days, then were transferred to PKUFH Experimental Animal Center. The rats were housed in wire mesh cages in an air-conditioned room (22 ± 2°C) on a 12-h light/dark cycle (06:00–18:00) and *ad libitum* access to food and water. The plateau group was preconditioned to a simulated altitude of 5,000 m (barometric pressure [PB], 405 mmHg; partial pressure of oxygen [PO_2_], 84 mm) for 6 h/day, for a total of 7 days. The plateau environment was stimulated by the high pressure/low pressure oxygen tank in The Sixth Medical Center of PLA General Hospital. We placed the rats of plateau group into the tank at same time each day (in light cycle) and provide them the same foods and temperature as outside. The plain group was exposed to ordinary atmospheric conditions (PB, 760 mmHg; PO_2_, 157 mmHg) for 7 days.

### Young-Rat Seizure Model

After preconditioning, all experimental animals received an intraperitoneal injection (i.p.) of 20 mg/kg (The dosage was a moderate dosage compared with those reported by other institutions) pentylenetetrazol (PTZ, Sigma, USA) to generate the convulsion model freely dissolved in saline (solution: 20 mg PTZ dissolved in 1 ml saline) (11, 12). Extracorporeal electroencephalogram (EEG) electrodes was placed on 16 rats of each group to monitor the seizure (which don't get the additional PTZ). Behavioral seizures were graded according to the Racine scale (13) as follows: stage 1—behavioral arrest with mouth/facial movements; stage 2—head nodding; stage 3—forelimb clonus; stage 4—rearing or hind limb extension; and stage 5—continuous rearing and falling and loss of balance. Rats were observed for 25 min after drug administration. If the seizures of the rest 84 rats were not severe enough to reach stage 4, an additional dose of 15 mg/kg (solution: 15 mg PTZ dissolved in 1 ml saline) was administered until the occurrence of a convulsive seizure enduring the duration of 5 min. The operation was repeated for up to 3 days; we remeasure the rats' weight daily before giving the drug. If the seizure reached grade 4 or 5 in 2 days, the model was defined as “successful” and the follow-up experiment was performed. If the seizure of any rats lasted for 10 min, 5% chloral hydrate was given to stop the attack (40–60 mg/kg, i.p., Sigma, USA).

### Brain Function Evaluation

#### Evaluation of Brain Electrical Signals

Randomly selected rats from the plateau and plain groups (16 rats per group), for all rat models, 15% chloral hydrate were administered 150 mg/kg intraperitoneally, then fixated on a stereotaxic apparatus. Cranial were exposed and drilled at bilateral frontal and temporal area. Bilateral frontal electrodes were placed 2 mm anterior and 2 mm lateral to the bregma. Bilateral temporal electrodes were placed 4 mm lateral to the bregma and as close to the ear as possible. Four stainless steel screws, 0.5 mm in diameter, were placed epidurally. Wirings were connected and fixed with self-curing denture acrylic. Aseptic techniques were observed during the whole procedure. Then the rats were administered PTZ and then were connected with a signal analysis system (BL-420N Biological, Signal Acquisition, and Analysis System) to monitor the brain electric signals at baseline—before the onset of convulsion—and signals in the middle and after the termination of convulsion, with a duration of 5 min each. The severe stage, duration, amplitude of convulsion, frequency of onset spike, interval of wave peak, and voltage suppression time after convulsion were recorded.

#### Histopathological Analysis of Acute Brain Injury

Six successful rats models were randomly selected from the plateau and plain groups, and brain tissue was collected at 24 h (3 rats per group) and 72 h (3 rats per group) after the last seizure. After receiving an i.p. injection of 10% chloral hydrate (600 mg/kg), rats were transcardially perfused with 0.9% NaCl followed by 4% phosphate-buffered saline (PBS). The brains were carefully removed and the tissue was fixed in PBS at 4°C for 72 h. The tissue was embedded in paraffin and cut into a series of 4-μm sections. A total of nine sections were examined per brain: three were stained with hematoxylin-eosin (HE) (Servicebio, G1005, Wuhan, China) for the study of cellular morphology; three were stained with cresyl violet (Nissl) to identify neuronal cells (200 × fields); and the remaining three were examined with the terminal deoxynucleotidyl transferase dUTP nick end labeling (TUNEL) assay to identify apoptotic cells.

The histological structure was observed under a light microscope (Nikon Eclipse E100, Japan). We chose the hippocampus because it is closely associated with memory and cognition, and is known to be actively involved in epileptogenesis, with CA1, CA3, and DG being the most representative and sensitive regions (14, 15). Three fields of vision were randomly selected from the CA1, CA3, and dentate gyrus (DG) areas of the hippocampus for each. The number of dark Nissl-stained neurons were counted. TUNEL-positive cells were counted with the Pannoramic viewer (3D HISTECH, Hungary), after the sections were scanned (Pannoramic MIDI, 3DHISTECH, Hungary) (16).

#### Evaluation of Spatial Learning and Memory

The Morris water maze was used to evaluate any spatial learning and memory differences between the plateau and plain groups. Twelve rats from each group were randomly selected at 6-weeks of age. The experiment was performed in a circular pool with a 120-cm diameter, 50-cm wall height, and 25-cm water depth. The water temperature was set at 26°C, and the color of the water was turned white with the use of a milk powder. The escape platform was placed in the second quadrant, 1 cm below the water surface. Three objects of different shapes were placed on the shore of the pool as spatial cues. The camera was placed directly above the pool, and the rat's head was dyed black before entering the water to facilitate the camera recording. Once rats were placed in the pool, they started swimming until they successfully found the hidden platform (success rate, 100%); if after 60 s they did not find the platform, they were removed from the pool (success rate, 0%). During the first 5 days of the experiment, known as the place navigation test, the rats' spatial learning and memory were analyzed. Each entry point was randomly selected from the edge of the pool. Each rat was tested five times per day for a total of 5 days. When a test finished, the rat stayed on the platform to rest for 30 s, and was then moved on to the next training session. The swimming distance traveled (cm), the escape latency to successfully find the hidden platform for the first time after each entry (s), the total distance swum in the platform quadrant (cm), the duration in the platform quadrant (s), and the total distance swum before reaching the platform for the first time (cm) were recorded and analyzed (17, 18). On the sixth day, rats underwent the reversal test at the same time as that during the first 5 days to investigate the rat's ability to retain spatial memory. For this, the platform was removed and placed at the point furthest from the original position. The number of crossings within the area where the original platform had been during a time interval of 120 s, the stay duration (s) and swimming distance (cm) in the original platform quadrant, the numbers of crossing the original platform quadrant, the total distance traveled (cm), and the time point (s) when the original platform was crossed for the first time were recorded.

### Statistical Analysis

Quantitative data are expressed as the mean ± standard error of the mean (SEM). The data were statistically analyzed using the SPSS 21.0 software. Independent sample *T*-test was used to compare the statistical difference of electroencephalogram parameters (duration, amplitude of convulsion, frequency of onset spike, interval of wave peak, and voltage suppression time after convulsion) and neuronal counts, analysis of variance for repeated data was used for parameters of Morris water maze (the escape latency, total distance traveled, distance spent in platform quadrant/total distance traveled, and platform quadrant duration/escape latency period). A value of *P* < 0.05 was considered as statistically significant.

## Results

### Seizure Manifestation and Electroencephalogram Parameters

After preconditioning, all rats were given PTZ to generate seizure models. After the end of seizures, the survival rate in the plateau group was significantly lower than in the plain group (26.2 vs. 42.9%, respectively; *p* < 0.05). The drug dose for inducing stage 4 or 5 seizures successfully was significantly higher in the plateau group compared to in the plain group (81.32 ± 21.57 vs. 63.41 ± 19.77 mg/kg, respectively; *p* < 0.01).

The baseline characteristic showed slow waves of 3–5 Hz in all rats in intermittent period. No differences in EEG signals during and after the seizures were observed between the plateau and the plain group (frequency of sharp wave: 5.78 ± 1.90 vs. 4.81 ± 1.88 Hz, respectively; *p* = 0.051). Similarly no differences between the two groups were observed in seizure duration and amplitude, interval of crest, and duration of post-seizure electrical suppression (Table 1).

**Table 1:** Electroencephalogram parameters in the plateau and plain groups. Values are the mean±SEM.

	**Plateau (*n* = 16)**	**Plain (*n* = 16)**	***p*-value**
Frequency of sharp wave (Hz)	5.78 ± 1.90	4.81 ± 1.88	0.051
Duration (s)	54.30 ± 51.29	65.87 ± 84.22	0.490
Amplitude (μV)	783.42 ± 247.40	830.17 ± 281.21	0.409
Interval of crest (s)	0.23 ± 0.99	0.18 ± 0.75	0.067
Duration of post-seizure electrical suppression(s)	8.19 ± 8.76	10.35 ± 7.25	0.316

### Histopathological Findings

**Figure 1:**
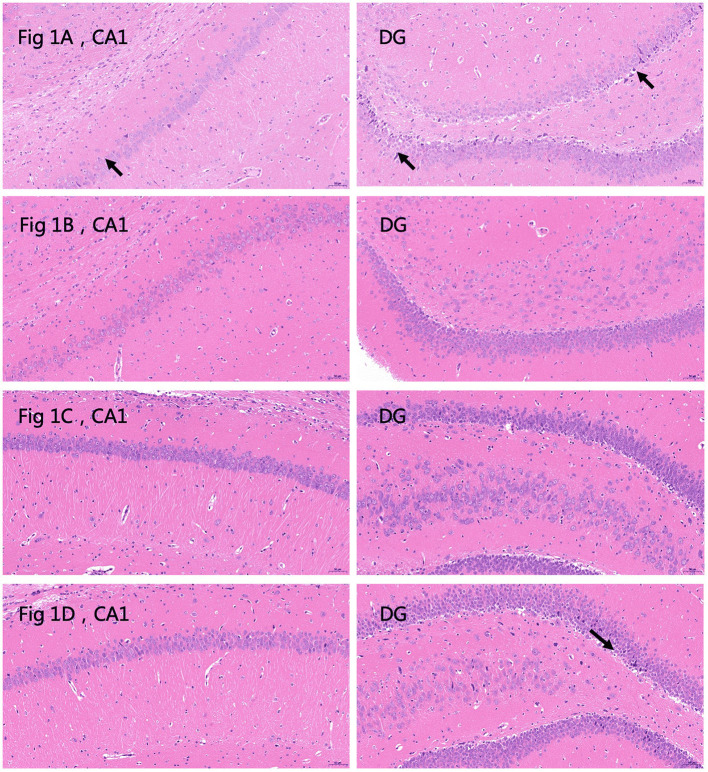
Hematoxylin and eosin (HE) stained sections depicting the CA1 and dentate gyrus (DG) areas of the hippocampus. **(A)** Plain group, 24 h after the last seizure. The arrows indicate deeply stained cells. Neurons are abundant and closely arranged with clear boundaries. A small number of pyramidal cells are deeply stained, and the boundaries between cytoplasm and nucleus are unclear (black arrow). No other obvious abnormalities are observed (20.0×). **(B)** Plain group, 72 h after the last seizure. Neurons are abundant and closely arranged, with clear boundaries and normal morphology. The boundaries between cytoplasm and nucleus are clear, and no obvious abnormalities are observed (20.0×). **(C)** Plateau group, 24 h after the last seizure. Neurons are abundant and regularly arranged, with normal morphology and structure of neurons, clear boundaries of cytoplasm and nucleus, and no obvious inflammation (20.0×). **(D)** Plateau group 72 h after the last seizure. The arrows indicate deeply stained cells. The number of neurons in the hippocampal CA1 region is abundant and regular, the morphology and structure of neurons are normal, and the boundaries of nuclei and cytoplasm are clear. A small number of neurons are wrinkled in the DG region, the cells are deeply stained, and the boundaries of the nuclei and cytoplasm are unclear (black arrow) (20.0×).

Histological examination of HE sections revealed that in the plain group, 24 h after the last seizure, CA1 and DG cell morphology were normal, except for a small amount of cytoplasm that was deeply stained and showed unclear cytoplasmic and nuclear boundaries (Figure 1A); 72 h after the last seizure the CA1 and DG hippocampal areas appeared normal (Figure 1B). In the plateau group, 24 h after the last seizure, CA1 and DG neuronal morphology appeared normal (Figure 1C); however, 72 h after the last seizure, a few neurons in the DG appeared wrinkled, the cell staining was darker, and the cytoplasmatic boundaries were unclear (Figure 1D). These results suggest that 72 h after the seizure cell morphology seems to be affected in the plateau but not the plain group.

**Figure 2:**
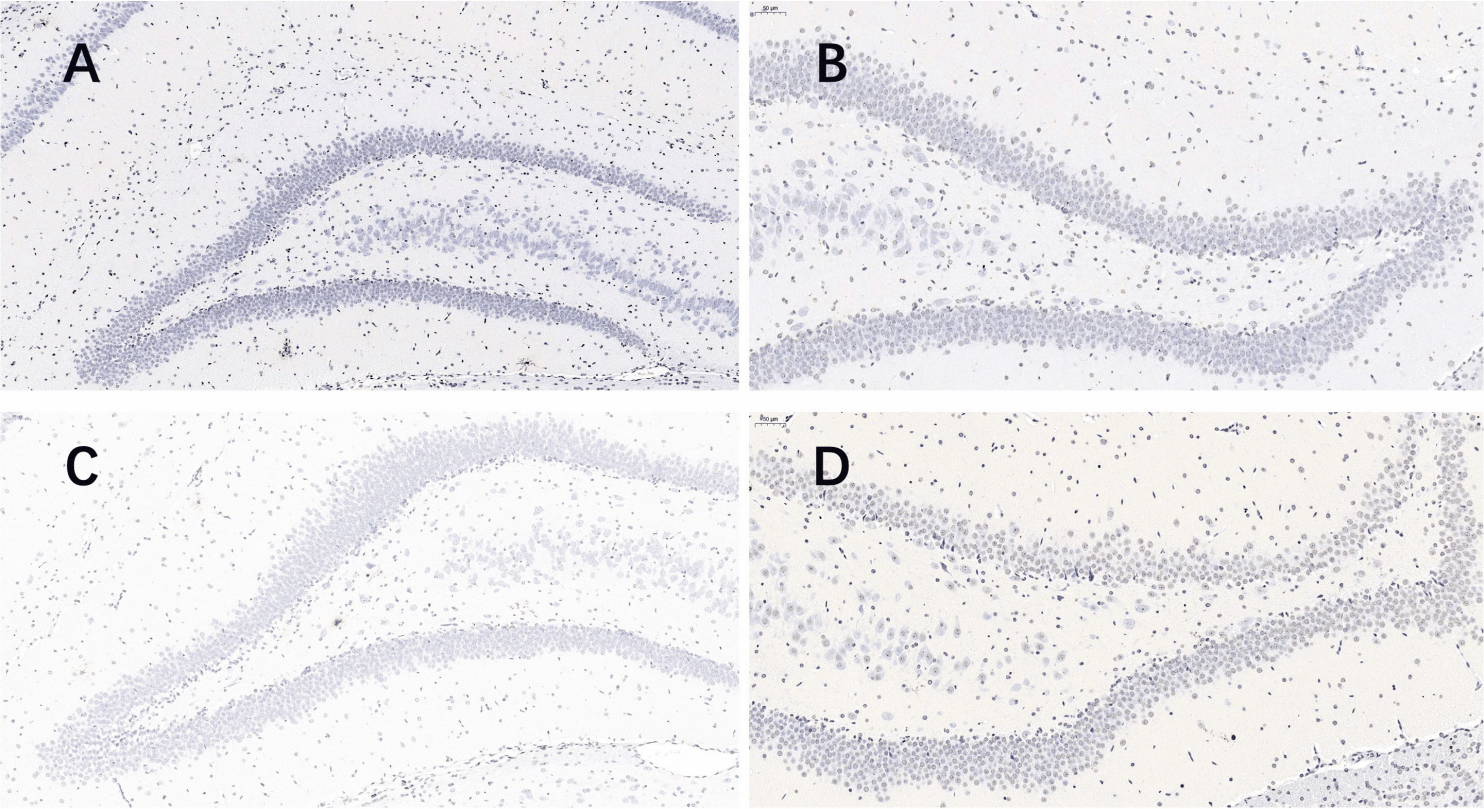
Nissl stained pictures of the hippocampus. When taking screenshots, try to fill the field of whole tissue, make sure that the background light of each photo is consistent. The nuclei of the surviving neurons were large and round, pale blue or blue, with clear nucleoli and abundant cytoplasm. **(A)** plateau group, 24 h after the last seizure; **(B)** plain group, 24 h after the last seizure; **(C)** plateau group, 72 h after the last seizure; **(D)** plain group, 72 h after the last seizure (20.0×).

Nissl staining (Figure 2) was used to evaluate neuronal survival in the brains of rats with seizures. Neuronal counts revealed a significant increase in the number of Nissl-positive cells in the DG of the plateau group compared to that in the plain group both at 24 and 72 h after the last seizure and in CA1 of the plateau group compared to that in the plain group at 72 h. No differences between groups were observed in the number of neuronal cells in the CA3 hippocampal areas (Table 2).

**Table 2:** Neuronal counts revealed by Nissl staining in the plateau and plain groups at 24 and 72 h after the last seizure.

		**Plateau**	**Plain**	***p*-value**
24 h	CA1	252.44 ± 35.15	264.33 ± 27.60	0.224
	CA3	255.00 ± 25.61	267.78 ± 19.97	0.279
	DG	946.33 ± 17.27	803.78 ± 24.49	<0.001
72 h	CA1	289.11 ± 15.15	206.78 ± 51.75	0.003
	CA3	270.56 ± 27.30	266.33 ± 31.90	0.827
	DG	954.78 ± 23.82	758.00 ± 85.77	<0.001

Values are the mean ± SEM.

*DG, dentate gyrus*.

**Figure 3:**
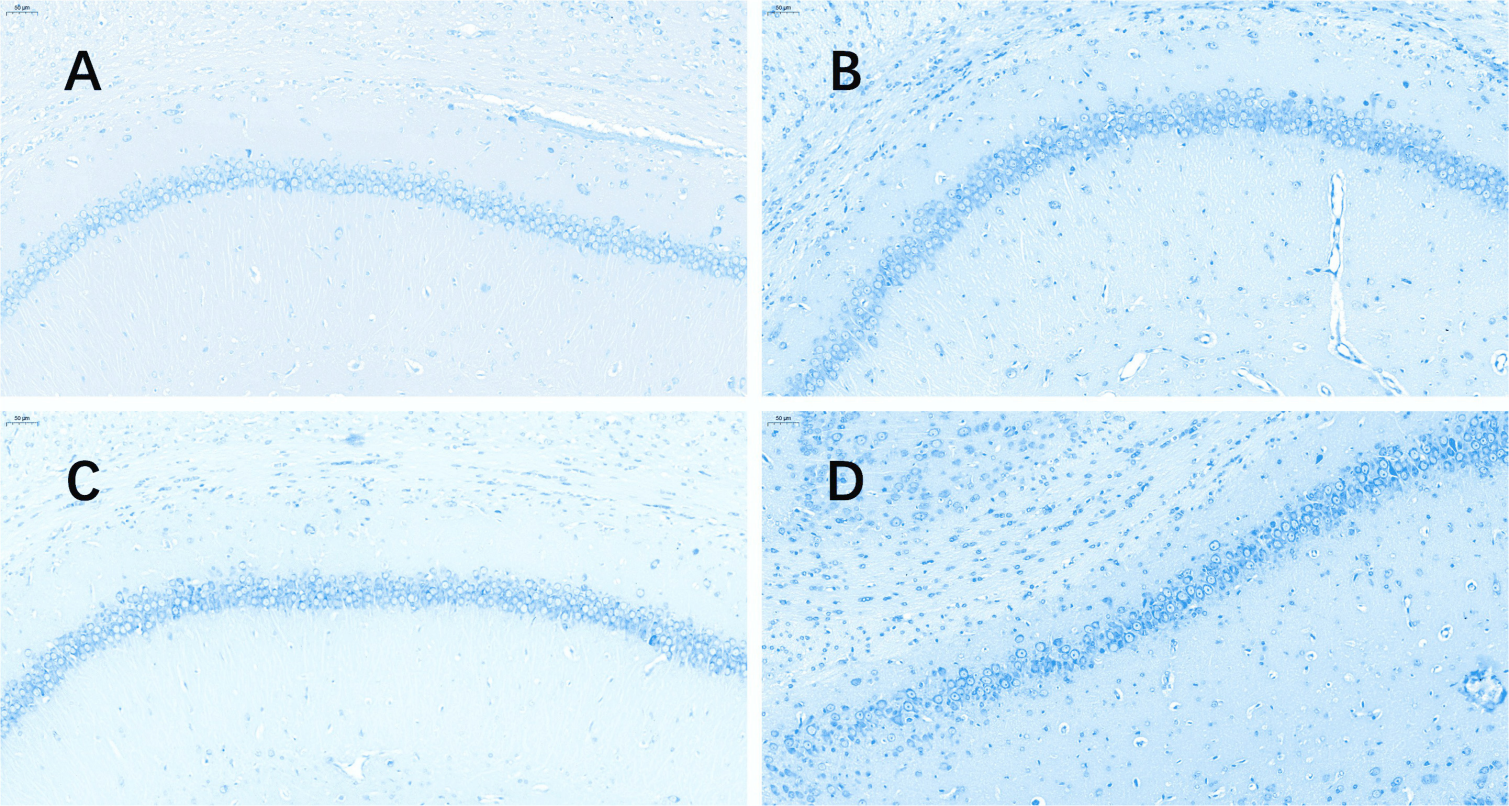
TUNEL stained pictures of the hippocampus. The software nuclei quant automatically identified all the neurons with dark brown, claybank, and light yellow nuclei as positive and blue nuclei as negative. The positive rate was the apoptosis rate (%). **(A)** Plateau group, 24 h after the last seizure; **(B)** plain group, 24 h after the last seizure; **(C)** plateau group, 72 h after the last seizure; **(D)** plain group, 72 h after the last seizure (20.0×).

The TUNEL (Figure 3) assay revealed a significant increase in hippocampal apoptotic cell death rate in the plain group of DG areas compared to that in the plateau group at 72 h after the last seizure (*p* = 0.009); however, no differences were observed in the apoptotic rates between the two groups at 24 h after the last seizure (Table 3).

**Table 3:** Apoptotic rates revealed by TUNEL staining in the plateau and plain groups at 24 and 72 h after the last seizure.

		**Plateau**	**Plain**	***p*-value**
24 h apoptotic rate (%)	CA1	51.19 ± 15.20	51.52 ± 8.64	0.485
	CA3	47.55 ± 27.75	57.13 ± 22.97	0.461
	DG	40.97 ± 24.05	50.04 ± 12.48	0.513
72 h apoptotic rate (%)	CA1	38.34 ± 25.14	68.38 ± 15.92	0.312
	CA3	45.24 ± 13.54	63.76 ± 15.39	0.342
	DG	39.88 ± 4.63	71.17 ± 8.45	0.035

*Values are the mean ± SEM*.

### Morris Water Maze Performance

In the place navigation test, the escape latency, total distance traveled, distance spent in platform quadrant/total distance traveled, platform quadrant duration/escape latency period (Table 4) were analyzed, and there were no statistical differences between the two group.

**Table 4:** Analysis of place navigation of the Morris water maze.

	**Escape Latency (s)**		**Total distance (cm)**		**Distance in platform**		**Platform quadrant**	
					**quadrant/total distance**		**duration/escape latency**	
	**Plateau**	**Plain**	**Plateau**	**Plain**	**Plateau**	**Plain**	**Plateau**	**Plain**
Day 1	38.81 ± 21.41	30.77 ± 22.49	8,207.20 ± 4,447.56	8,031.70 ± 5123.23	0.32 ± 0.13	0.31 ± 0.14	0.37 ± 0.17	0.46 ± 0.32
Day 2	24.57 ± 20.42	27.56 ± 20.21	5,387.09 ± 4,463.94	6,547.84 ± 5198.70	0.39 ± 0.19	0.30 ± 0.16	0.49 ± 0.28	0.33 ± 0.18
Day 3	25.05 ± 20.66	22.80 ± 16.07	5,296.64 ± 4,018.47	6,275.21 ± 3974.32	0.31 ± 0.19	0.32 ± 0.13	0.39 ± 0.23	0.50 ± 0.35
Day 4	22.86 ± 17.71	23.27 ± 21.18	4,764.52 ± 3,378.02	5,143.16 ± 4653.78	0.35 ± 0.19	0.32 ± 0.21	0.44 ± 0.27	0.39 ± 0.26
Day 5	22.39 ± 20.17	25.83 ± 18.85	4,740.10 ± 3,867.63	5,866.05 ± 3909.33	0.29 ± 0.14	0.33 ± 0.13	0.36 ± 0.23	0.39 ± 0.20
*F*	0.053		0.968		0.233		0.019	
*P*-value	0.818		0.330		0.631		0.890	

*Values are the mean ± SEM*.

In the reversal phase of the experiment on day 6, there was no statistical difference between the two groups in the number of crossings around the area of the original platform, total distance traveled, swimming distance in the original platform quadrant/total distance, and the number of times entering the platform quadrant (Table 5).

**Table 5:** Analysis of the reversal phase of the Morris water maze.

	**Plateau**	**Plain**	***p*-value**
Number of crossings through the original platform	1.57 ± 0.79	1.00 ± 1.22	0.345
Number of entering the platform quadrant	6.28 ± 1.60	5.60 ± 1.82	0.505
Total distance (cm)	12,200.98 ± 976.21	12,290.79 ± 1,395.34	0.898
Swimming distance in the original platform quadrant/total distance	0.33 ± 0.06	0.31 ± 0.11	0.874

*Values are the mean ± SEM*.

## Discussion

Plateau areas are a special survival and living environment. Plateaus' hypoxic and hypobaric conditions can produce complex overall responses from the various physiological functions of the human body. This, in turn, has led to differences in the disease spectra, disease characteristics, and prognosis between populations in Tibet and the plains. Convulsions are abnormal movements, behaviors, and autonomic nervous system functions, caused by the excessive depolarization and abnormal synchronous discharge of neurons. During infancy, the particularity of neurotransmitter development and the susceptibility of immature brain tissue to injury imply that the incidence of convulsions in this period is higher than that in children of other age groups and adults. Convulsions are not only the most common symptom of neurological injury, but can also lead to brain damage, residual epilepsy, developmental delay, other neurological sequelae, and even death in severe cases, affecting the patient's prognosis. However, existing research findings on convulsions and convulsive brain injury are all based on clinical data of pediatric patients from plain areas, whereas the incidence and prognosis of convulsions in children from plateau areas are still unclear. There is a lack of clinical research data on the effects of hypoxia and hypobaria on children's brain functions after convulsions. This is because, on one hand, Tibet covers a wide area and is sparsely populated, which makes it difficult to collect follow-up data. On the other hand, patient prognosis is affected by various factors such as whether medical attention was sought in time and the cause of convulsions. Thus, it is difficult to reflect on the role of hypoxic and hypobaric environments in the prognosis of infantile convulsions based on clinical follow-up alone. For these reasons, this study performed an animal experiment to investigate the onset and prognosis of seizures in young rats under a plateau environment, in order to provide a theoretical basis for the clinical treatment of pediatric patients with convulsions in plateau areas, and to clarify whether hypoxia and hypobaria have possible protective effects on the brain in pediatric patients with convulsions.

With respect to the occurrence of induced convulsions, stage 4–5 seizures were induced in 84 young rats each in the plateau and plain groups. Regarding the drug dose needed for the successful induction of stage 4–5 seizures in young rats, the plateau group required a significantly higher dose than the plain group, that means with the same PTZ dosage, less rats in plateau group would reach stage 4–5 seizures compared with the plain group, in other words, the threshold for convulsion in the plateau group was higher than that in the plain group. This indicates that preconditioning with the hypobaric and hypoxic conditions of the plateau environment may increase an individual's threshold to convulsions and make them less prone to the occurrence of convulsions. Hypobaric and hypoxic preconditioning has now been applied to the prevention and treatment of diseases that may cause organ hypoxia and ischemia, such as respiratory failure, coronary heart disease, and stroke. The underlying mechanism is related to the combined actions of multiple factors and signaling pathways, such as the involvement of glial glutamate transporter-1 (GLT-1) (19), heat-shock protein 70 (HSP70) (20), nitric oxide synthase (NOS) (21), and the Bcl-2 (22) family in the apoptotic pathway. Our study suggests that hypobaric and hypoxic environments may play a role in protecting the damage of convulsions, but the specific mechanisms that underline this will need to be verified by further basic research.

In terms of the short-term prognosis of convulsions in young rats in a plateau environment, we found that although the threshold of the drug dose for inducing convulsions was higher in this group, upon the induction of seizures, the mortality rate of young rats in the acute stage was significantly higher than the plain group. One of the mechanisms by which convulsions cause brain damage and even death is loss of oxygen (23), the results of some previous studies suggest that hypobaric and hypoxic preconditioning can improve the body's tolerance, and increase the tolerance of organs to the re-occurrence of hypoxia (9, 24). However, other studies have differing views, supporting instead that whether hypoxic preconditioning is damaging or protective is mainly determined by the degree of hypoxia (9). Our experimental results indicate that the dosage to induce severe convulsion in plain group is not enough to induce the same stage convulsion in plateau group, young rats that died following severe stage 4–5 seizures were critically ill. In such cases, even prior hypobaric and hypoxic preconditioning could not reverse disease progression, but instead it might cause serious consequences due to the underlying hypobaric and hypoxic state. We supposed maybe the tolerance increase in a certain range, once breakthrough the threshold, the ability to protect and compensate is broken, there is a cliff-like mechanism leading to a high mortality rate. The causes of death from severe epilepsy are very complex, including cerebral edema, respiratory arrest (25), capillary telangiectasias in the hippocampus (26), damage to other organs, and some unclear causes. According to the results of our research, we speculate that the reason lead to the high mortality with the low neuronal apoptosis rate in the plateau group may be that the hypobaria and hypoxia environment actually protects the neurons, but it does not prevent the other causes that directly cause the death. But the mechanism is only a hypothesis, which needs to be verified in future studies. Although the mortality rate of young rats in the plateau preconditioning group was higher than that in the plain group, rats that survived after seizure induction showed lower neuronal apoptosis in the acute stage than the plain group, while the neuronal survival rate in the DG at both time points and in the CA1 at 72 h was higher than that in the plain group. These observations indicate that for young rats with relatively mild conditions, hypobaria and hypoxia may still have a certain protective effect on the brain in the acute stage.

With regards to long-term prognosis, our water maze findings indicate that the performance of the young rats in the plateau and plain groups are comparable. This reflects, to a certain extent, that although the preconditioning environment of the plateau group did not improve the prognosis of the neurological development of young rats with convulsions, it also did not aggravate the impact of brain injury on their learning and memory abilities. The degree of convulsions induced in this study was severe, and the early mortality rate was high. Hence, the protective effect of hypobaric and hypoxic preconditioning in critical cases was not apparent. However, for surviving young rats with relatively mild conditions, hypobaria, and hypoxia might have exerted protective effects on the brain, which were manifested as increased neuronal survival rate in some brain regions and decreased neuronal apoptosis. In future studies, we could attempt to induce relatively mild seizures in young rats and evaluate their long-term prognosis, to investigate the protective effects of hypobaric and hypoxic preconditioning in young rats with mild seizures.

There are limitations to this study. We investigated the incidence and prognosis of young rats with convulsions by preconditioning them to a simulated altitude of 5,000 m with intermittent hypobaria and hypoxia, however, residents of Tibet live at an average altitude of ~4,000 m and are in a continuous state of hypobaria and hypoxia, experimental conditions that more closely simulate the real environment should be designed in the future to further explore the incidence and neurological prognosis of infantile convulsions in plateau areas. Secondly, because the drug dose in two groups are different, this may be one of the reason attribute to the high mortality rate, but since the induced convulsions stages were ultimately the same, we think our results are credible, but more studies on the dose-effect relationship would be helpful to eliminate the dosage bias. Last but not least, in this manuscript, we mainly described the phenomena we discovered, which we thought could be attributable to a neuro-protective effect, but confirmation as well as further research into the mechanism is needed.

## Conclusion

Our results suggest that the effect of the plateau environment on young rats with convulsive brain injury may be related to their disease severity. For individuals in critical condition, hypobaria and hypoxia are factors that will aggravate their poor prognosis, whereas for normal individuals or those with mild conditions, hypobaria and hypoxia may have a protective effect against convulsive brain injury. However, the specific mechanisms involved still require further investigation.

## Data Availability Statement

The raw data supporting the conclusions of this article will be made available by the authors, without undue reservation.

## Ethics Statement

The animal study was reviewed and approved by Peking University First Hospital Ethics Committee.

## Author Contributions

YX wrote the manuscript and conducted the statistical analysis. SQ and RZ conducted the experiments with the help of GS, under the supervision of LL, XH, and HW. All authors contributed to the article and approved the submitted version.

## Conflict of Interest

The authors declare that the research was conducted in the absence of any commercial or financial relationships that could be construed as a potential conflict of interest.

## References

[1] Johnston MV Hypoxic-ischemic encephalopathy. Am J Perinatol. (2000) 17:113–20. 10.1055/s-2000-9293 11012134

[2] Serebrovskaya TV. Intermittent hypoxia research in the former Soviet Union and the commonwealth of independent States: history and review of the concept and selected applications. High Alt Med Biol. (2002) 3:205. 10.1089/15270290260131939 12162864

[3] Goryacheva AV Kruglov SV Pshennikova MG Smirin BV Malyshev IY Barskov IV. Adaptation to intermittent hypoxia restricts nitric oxide overproduction and prevents beta-amyloid toxicity in rat brain. Nitric Oxide. (2010) 23:289–99. 10.1016/j.niox.2010.08.005 20804853

[4] Rybnikova E Samoilov M. Current insights into the molecular mechanisms of hypoxic pre- and postconditioning using hypobaric hypoxia. Front Neurosci. (2015) 9:388. 10.3389/fnins.2015.00388 26557049 PMC4615940

[5] Silverstein FS Jensen FE. Neonatal seizures. Ann Neurol. (2007) 62:112–20. 10.1002/ana.21167 17683087

[6] Tekgul H Gauvreau K Soul J Murphy L Robertson R Stewart J. The current etiologic profile and neurodevelopmental outcome of seizures in term newborn infants. Pediatrics. (2006) 117:1270–80. 10.1542/peds.2005-1178 16585324

[7] Henshall DC Murphy BM. Modulators of neuronal cell death in epilepsy. Curr Opin Pharmacol. (2008) 8:75–81. 10.1016/j.coph.2007.07.005 17827063

[8] Dirnagl U Becker K Meisel A. Preconditioning and tolerance against cerebral ischaemia: from experimental strategies to clinical use. Lancet Neurol. (2009) 8:398–412. 10.1016/S1474-4422(09)70054-7 19296922 PMC2668955

[9] Zhen JL Wang WP Zhou JJ Qu ZZ Fang HB Zhao RR. Chronic intermittent hypoxic preconditioning suppresses pilocarpine-induced seizures and associated hippocampal neurodegeneration. Brain Res. (2014) 1563:122–30. 10.1016/j.brainres.2014.03.032 24680745

[10] Li Q Han Y Du J Jin H Zhang J Niu M. Alterations of apoptosis and autophagy in developing brain of rats with epilepsy: Changes in LC3, P62, Beclin-1 and Bcl-2 levels. Neurosci Res. (2018) 130:47–55. 10.1016/j.neures.2017.08.004 28807642

[11] Lüttjohann A Fabene PF van Luijtelaar G. A revised Racine's scale for PTZ-induced seizures in rats. Physiol Behav. (2009) 98:579–86. 10.1016/j.physbeh.2009.09.005 19772866

[12] Hussein AM Eldosoky M El-Shafey M El-Mersery M Abbas KM Ali AN. Effects of GLP-1 receptor activation on a pentylenetetrazole-kindling rat model. Brain Sci. (2019) 9:108. 10.3390/brainsci9050108 31091715 PMC6562858

[13] Racine RJ. Modification of seizure activity by electrical stimulation. II. Motor seizure. Electroencephalogr Clin Neurophysiol. (1972) 32:281–94. 10.1016/0013-4694(72)90177-0 4110397

[14] Zhvania MG Ksovreli M Japaridze NJ Lordkipanidze TG. Ultrastructural changes to rat hippocampus in pentylenetetrazol- and kainic acid-induced status epilepticus: a study using electron microscopy. Micron. (2015) 74:22–9. 10.1016/j.micron.2015.03.015 25978010

[15] Bliss TV Collingridge GL. A synaptic model of memory: long-term potentiation in the hippocampus. Nature. (1993) 361:31–9. 10.1038/361031a0 8421494

[16] Li Q Han Y Du J Jin H Zhang J Niu M. Recombinant human erythropoietin protects against hippocampal damage in developing rats with seizures by modulating autophagy via the S6 protein in a time-dependent manner. Neurochem Res. (2018) 43:465–76. 10.1007/s11064-017-2443-1 29238892

[17] Vorhees CV Williams MT. Morris water maze: procedures for assessing spatial and related forms of learning and memory. Nat Protoc. (2006) 1:848–58. 10.1038/nprot.2006.116 17406317 PMC2895266

[18] Schoenfeld R Schiffelholz T Beyer C Leplow B Foreman N. Variants of the Morris water maze task to comparatively assess human and rodent place navigation. Neurobiol Learn Mem. (2017) 139:117–27. 10.1016/j.nlm.2016.12.022 28057502

[19] Gong SJ Chen LY Zhang M Gong JX Ma YX Zhang JM. Intermittent hypobaric hypoxia preconditioning induced brain ischemic tolerance by up-regulating glial glutamate transporter-1 in rats. Neurochem Res. (2012) 37:527–37. 10.1007/s11064-011-0639-3 22076500

[20] Lin HJ Wang CT Niu KC Gao C Li Z Lin MT. Hypobaric hypoxia preconditioning attenuates acute lung injury during high-altitude exposure in rats via up-regulating heat-shock protein 70. Clin Sci. (2011) 121:223–31. 10.1042/CS20100596 21599636

[21] Huang YJ Yuan YJ Liu YX Zhang MY Zhang JG Wang TC Nitric oxide participates in the brain ischemic tolerance induced by intermittent hypobaric hypoxia in the hippocampal CA1 subfield in rats. Neurochem Res. (2018) 43:1779–90. 10.1007/s11064-018-2593-9 29995175

[22] Wu Q Yu KX Ma QS Liu YN. Effects of intermittent hypobaric hypoxia preconditioning on the expression of neuroglobin and Bcl-2 in the rat hippocampal CA1 area following ischemia-reperfusion. Genet Mol Res. (2015) 14:10799–807. 10.4238/2015.September.9.18 26400308

[23] Björkman ST Miller SM Rose SE Burke C Colditz PB. Seizures are associated with brain injury severity in a neonatal model of hypoxia-ischemia. Neuroscience. (2010) 166:157–67. 10.1016/j.neuroscience.2009.11.067 20006975

[24] Cuomo O Vinciguerra A Cerullo P Anzilotti S Brancaccio P Bilo L. Ionic homeostasis in brain conditioning. Front Neurosci. (2015) 9:277. 10.3389/fnins.2015.00277 26321902 PMC4530315

[25] Kommajosyula SP Randall ME Brozoski TJ Odintsov BM Faingold CL. Specific subcortical structures are activated during seizure-induced death in a model of sudden unexpected death in epilepsy (SUDEP): a manganese-enhanced magnetic resonance imaging study. Epilepsy Res. (2017) 135:87–94. 10.1016/j.eplepsyres.2017.05.011 28646692

[26] Liu Y Liang Y Tong F Huang W Tinzing L Le Grange JM. Sudden death from an epileptic seizure due to capillary telangiectasias in the hippocampus. Forensic Sci Med Pathol. (2019) 15:243–8. 10.1007/s12024-018-0075-7 30649694

